# Genetic and epidemiological analyses of alcohol consumption patterns and age-related macular degeneration risk among current drinkers: a role for TNFRSF10A

**DOI:** 10.7189/jogh.16.04124

**Published:** 2026-05-22

**Authors:** Ting Shen, Jing Li, Xiongyi Yang, Jiao Xia, Han Zhou, Qian Ma, Yuling Wang, Jiajia Wang, Zhenhua Wang, Kun Liu, Biao Yan

**Affiliations:** 1 Department of Ophthalmology, Shanghai General Hospital, Shanghai Jiao Tong University School of Medicine, Shanghai, China; 2 Health and Human Sciences, Faculty of Medicine, Macquarie University, Sydney, Australia; 3 Save Sight Institute, University of Sydney, Sydney, Australia; 4 Eye Institute and Department of Ophthalmology, Eye & ENT Hospital, Fudan University, Shanghai, China; 5 Department of Ophthalmology, General Hospital of Ningxia Medical University, Yinchuan, China; 6 College of Information Science, Shanghai Ocean University, Shanghai, China

## Abstract

**Background:**

Alcohol intake, a major modifiable lifestyle factor, has been variably associated with age-related macular degeneration (AMD). We aim to investigate the association and potential causal relevance of alcohol consumption with AMD risk using complementary epidemiological and genetic approaches.

**Methods:**

We used nonlinear Cox proportional hazards models to evaluate incident AMD associations among 440 052 participants of European ancestry with a mean age of 56.7 years and of whom 53.7% were female. We employed two-sample Mendelian randomisation (MR), integrating summary-level genetic associations with cis-expression quantitative trait locus colocalisation between alcohol consumption (UK Biobank n = 433 353) and AMD (GWAS Catalog n = 105 248; FinnGen study n = 474 181), to refine genetic instruments for biologically relevant measures of alcohol consumption. We also used inverse-variance weighting (IVW) and conducted sensitivity analyses. In addition, we integrated publicly available single-cell RNA sequencing (scRNA-seq) data from human retina and retinal pigment epithelium/choroid tissues to assess cell-type-specific expression patterns of genes implicated in AMD.

**Results:**

Over the follow-up period, 1553 participants were diagnosed with AMD. Alcohol consumption was inversely associated with AMD risk (hazard ratio (HR) = 0.83; 95% confidence interval (CI) = 0.73 − 0.95), with the association being most pronounced for wine (HR = 0.86; 95% CI = 0.76 − 0.97). Beer consumption showed a positive association with AMD risk. These results were supported by MR analyses (IVW *β* = −0.13; *P* = 0.02) and remained consistent in sensitivity analyses. Colocalisation analysis highlighted TNFRSF10A (PP.H4 = 0.98) as a shared locus associated with both alcohol intake and AMD risk. Integration with scRNA-seq data from human samples suggested endothelial cell-enriched expression of TNFRSF10A in AMD tissues, providing functional context for the genetic prioritisation of this locus.

**Conclusions:**

Epidemiological and genetic data suggest that TNFRSF10A and its variants may be involved in AMD, with alcohol consumption patterns potentially contributing to this association. This provides new hypotheses for how lifestyle factors may influence disease risk. These findings should not be interpreted as evidence supporting alcohol consumption for AMD prevention, given the potential for residual confounding and the well-established health risks of alcohol.

As individuals age, the influence of lifestyle on health becomes increasingly evident. Notably, alcohol consumption has drawn particular attention due to its potential role in the development of age-related diseases [1]. Targeted intervention in later life addressing this factor could improve overall public health outcomes. Age-related macular degeneration (AMD) is a leading cause of vision loss in the elderly and is often used as a model to study how lifestyle factors impact disease development [2,3]. However, the association between alcohol consumption and AMD risk remains controversial. Some studies suggest that alcohol accelerates AMD progression [4,5], while others report minimal effects [6–8] or even a potential protective effect [9]. These conflicting findings may reflect method limitations, including confounding and selection bias in study populations [7,9,10]. The mechanisms through which alcohol affects AMD remain poorly understood. Further investigation using approaches that can address confounding and clarify potential underlying biological links is needed to inform more effective prevention and management strategies.

Recent genomic studies have identified over 100 genetic variants associated with AMD, most of which are in non-coding regions, and their roles in gene regulation and pathogenesis remain unclear [11–13]. As a result, traditional epidemiological studies, limited by confounding factors and reverse causality, face challenges in establishing biologically meaningful links between genetic variation and AMD pathology using conventional approaches alone. Mendelian randomisation (MR) analysis, a causal inference method based on genetic variants, has been widely used to explore the causal relationship between environmental and lifestyle factors (such as alcohol consumption) and AMD risk [14]. A previous MR study suggested that any genetically predicted alcohol consumption was associated with an increased risk of geographic atrophy, a subtype of advanced AMD [15]. Individual differences in alcohol metabolism, cumulative exposure, and diverse drinking patterns suggest that the association between alcohol consumption and AMD may be nonlinear; lower levels of intake may be associated with reduced risk, whereas very high consumption may increase disease risk [16].

In this study, we aimed to investigate the association and potential causal relevance of alcohol consumption with AMD risk among current drinkers, using complementary epidemiological and genetic approaches, with a focus on beverage-specific patterns and the contribution of shared genetic variation.

## METHODS

### Study population and AMD definition

We obtained participant-level data from the prospective UK Biobank cohort, including baseline age, sex, and detailed alcohol intake indicators. In this population-based cohort study, we defined incident AMD as cases in which the recorded age at first diagnosis exceeded the age at baseline assessment (Figure S1 and Table S2 in the **Online Supplementary Document**). We restricted the primary analysis to current drinkers to reduce the potential influence of sick-quitter bias, a recognised issue in alcohol epidemiology in which former drinkers who abstain due to poor health can distort risk estimates if combined with lifelong abstainers [17,18].

### Outcome measurement

We extracted baseline variables, including age at recruitment, body mass index, physical activity, macular thickness, and socioeconomic status. We calculated the follow-up time from baseline to the earliest of loss to follow-up, AMD diagnosis, death, or 12 November 2024. We classified socioeconomic status using the Townsend Deprivation Index quantiles. We categorised physical activity as additional, sufficient, or insufficient based on reported intensity. Education level was categorised as ‘Higher or vocational’, ‘Upper or lower secondary’, or ‘Other’.

### Alcohol consumption measurements and nonlinear analyses

We calculated the total weekly alcohol intake (units/week) by summing self-reported consumption of red wine, white wine or champagne, beer or cider, spirits, fortified wine, and other alcoholic beverages for all current drinkers. We conducted subgroup analyses by alcohol type: wine (red, white, and champagne) and beer. However, we excluded spirits and other alcoholic beverages since they did not meet the proportional hazards assumption in multiple models. We excluded extreme outliers in weekly alcohol consumption (>40 units/week for total intake and >30 units/week for subgroups), retaining only plausible values within the observed distribution for the final analyses, as AMD cases were rare among individuals with very high alcohol intake. In sex-stratified sensitivity analyses, we did not perform subgroup assessments for beer and wine because the number of individuals with high intake (≥10 units/week) in each sex was limited. We also excluded participants with missing or non-numeric values.

### Multimodal data integration using summary-databased and two-sample MR

We integrated cis-expression quantitative trait loci (cis-eQTL) summary data from the eQTLGen consortium [19] with genome-wide association study (GWAS) summary statistics for alcohol consumption among current drinkers from the UK Biobank (GCST90132924) [15] using summary-databased MR (SMR) [20] (Supplement Methods in the **Online Supplementary Document**). We repeated the same procedure for AMD GWAS data (GCST010723) [12] to determine whether variants colocalised with drinking behaviour also showed pleiotropic effects in AMD. We used two-sample MR (TSMR) analysis, including the inverse-variance-weighted method, for the variants that passed the false discovery rate threshold to further evaluate potential causal effects.

### Functional enrichment and annotation analysis

We conducted gene ontology and pathway enrichment analyses on the identified genes [21,22]. We used functional mapping and annotation of GWAS to prioritise, visualise, and interpret the functions of genes mapped from single-nucleotide polymorphisms (SNPs) selected using instrumental variables from the TSMR analysis [23].

### Single-cell expression profiling and integrated analysis

We obtained single-cell RNA sequencing data from the Gene Expression Omnibus repository (GSE137537 and GSE135922) [24–26]. We subsequently created a Seurat object using CreateSeuratObject and incorporated metadata fields (*e.g.* cell type and tissue) from the sample annotation file. Since the data sets were publicly available and the samples were already annotated, we did not perform additional quality control. We independently normalised each data set using Seurat's LogNormalize method to establish a consistent analytical baseline within each study.

### Optical coherence tomography-based evaluation of macular thicknesses by SNP carriage

We evaluated the associations between three SNPs (rs541862, rs1265094, and rs3138141), each present in over 100 participants, and retinal measurements, including overall macular thickness, overall averaged retinal pigment epithelium (RPE) thickness, and macular central subfield thickness obtained from optical coherence tomography (OCT) (Topcon 3D OCT 1000 Mk2) in the UK Biobank cohort.

### Colocalisation, protein structure, candidate drug prediction, and molecular docking

We performed colocalisation analysis using the coloc.abf function, with a focus on posterior probabilities (H0–H4). We considered SNPs with PP.H4 > 0.8 colocalised. We obtained the predicted structure of TNFRSF10A using AlphaFold, version 2.3.2 [27]. We mapped drug target genes identified from the TSMR instrumental variables using the Drug Signatures Database, and retrieved protein structures from the Protein Data Bank and compound structures from PubChem. We simulated protein-ligand binding using AutoDock Vina, version 1.2.2. We assessed binding affinity using binding energy (kcal/mol), considering values<–5.0 as good and<–7.0 as strong binding.

### Statistical methods

We reported continuous variables as mean (standard deviation) or median (interquartile range), and categorical variables as counts and percentages. We used standardised mean differences to assess group imbalances. We used original data sets from the analyses without imputing missing values. We examined nonlinear associations between alcohol consumption and AMD risk using restricted cubic spline Cox models with three knots (at the 10%, 50%, and 90% quantiles), selected based on likelihood ratio tests. We confirmed the proportional hazards assumptions with Schoenfeld residuals. We adjusted the models for sex and used age at AMD diagnosis as the time scale to account for strong age effects, noting potential biases such as left truncation [28].

We used *R*, version 4.4.2 (R Core Team, Vienna, Austria) for all analyses. All tests were two-sided, with *P* < 0.05 considered significant.

## RESULTS

### Differential associations of alcohol type with AMD risk

We summarised baseline characteristics by sex (Table S3 in the **Online Supplementary Document**). Between males and females, standardised mean differences indicated generally small to moderate differences across key covariates. Body mass index and Townsend Deprivation Index exhibited slightly larger variability, suggesting possible differences in adiposity and socioeconomic conditions. We performed univariate Cox regression analyses to assess the independent associations of key potential confounders with AMD risk. Although specific categories of sleep duration (≥9 hours) and smoking status (current smokers) were associated with altered risk, the overall patterns for education, socioeconomic status, lifestyle factors, and comorbidities did not demonstrate strong, consistent independent associations with AMD risk (Table S4 in the **Online Supplementary Document**).

Subsequently, we constructed a multivariable Cox model to examine the association between alcohol consumption and AMD, adjusting for all these potential confounders plus sex (Table S4 in the **Online Supplementary Document**). None of the covariates materially altered the association between alcohol intake and AMD risk, and there were no significant interactions. Therefore, we adjusted the subsequent primary analyses of the alcohol-AMD relationship only for sex. Restricted cubic spline Cox analyses showed that total alcohol intake was associated with a modest reduction in AMD risk (hazard ratio (HR) = 0.83; 95% confidence interval (CI) = 0.73–0.95) (**Figure 1**; Table S5 in the **Online Supplementary Document**). The proportional hazards assumption was met (*P* = 0.16). Wine consumption showed a similar lower risk (HR = 0.86; 95% CI = 0.76–0.97), whereas beer intake was linked to an increased risk (HR = 1.20; 95% CI = 1.08–1.33) and displayed evidence of a nonlinear relationship (*P* = 0.006). The direction and magnitude of associations were largely consistent in gender-specific analyses (Figure S2, Panels A and B; Table S5 in the **Online Supplementary Document**).

Furthermore, we conducted interaction analyses to assess whether the association between alcohol consumption and AMD risk was modified by sex, smoking, sleep, body mass index, comorbidities, education, or socioeconomic status. None of these interaction terms were statistically significant (*P* range = 0.11–0.99) (Table S4 in the **Online Supplementary Document**), indicating that the observed alcohol-AMD associations were consistent across different levels of these potential modifiers. These findings suggest that the type of alcoholic beverage may affect AMD risk differently, with wine appearing inversely associated and beer potentially increasing the risk

### Integrative GWAS and eQTL analysis reveals genetic determinants of alcohol consumption

After harmonisation and frequency filtering, 7 119 893 common variants remained from an initial set of 10 150 997 GWAS and 8 932 843 eQTL SNPs. A total of 15 643 probes, each containing at least one cis-eQTL SNP (*P* < 5.00 × 10^−8^), were subjected to SMR analysis. The heterogeneity-in-dependent-instruments test excluded SNPs likely influenced by linkage disequilibrium, and false discovery rate correction identified multiple SNP-probe pairs with strong evidence of colocalisation. These results suggest a shared genetic basis linking genetically regulated gene expression and current alcohol consumption behaviour. Only a small subset of variants exhibited concordant eQTL and GWAS signals, supporting the use of SMR and colocalisation analyses to prioritise shared loci. We summarised the genome-wide distribution of gene-based association signals derived from GWAS summary statistics using multi-marker analysis of genomic annotation (Figure S3, Panels A–C in the **Online Supplementary Document**). Replication in the AMD data set identified a subset of 581 colocalised variants that also reached significance in SMR and heterogeneity in dependent instruments testing. These results suggest potential pleiotropic effects, implicating gene-expression-mediated pathways in both current alcohol consumption and AMD risk.

Using genes mapped from 581 SNPs associated with both alcohol consumption and AMD, gene ontology enrichment analysis identified key biological processes, cellular components, and molecular functions implicated in disease pathogenesis. In the biological processes category, enriched terms included regulation of T-cell, leukocyte, and mononuclear cell proliferation, highlighting the importance of immune regulatory pathways. Molecular functions analysis showed enrichment for metal cluster binding and iron-sulphur cluster binding, which are critical for oxidative stress regulation and mitochondrial function. In the cellular components domain, terms such as the luminal side of the endoplasmic reticulum membrane and ER-to-Golgi transport vesicle membranes were enriched, suggesting potential disruptions in protein transport and antigen presentation. Reactome pathway analysis identified enrichment of immune-related pathways among genes prioritised from the genetic analyses (Figure S4, Panels A–D in the **Online Supplementary Document**).

Using functional mapping and annotation, we annotated genes mapped from the prioritised SNPs and observed enrichment of apoptosis-related signalling pathways. Among these, CASP10, CFLAR, and TNFRSF10A showed broad expression across multiple tissues based on GTEx data (Figure S3, Panel D and Figure S4, Panel E in the **Online Supplementary Document**), supporting their biological plausibility as pathway-level candidates rather than tissue-specific signals, and highlighting their potential relevance to immune and apoptotic pathways implicated in AMD. Prioritised genes showed consistent overlap across apoptosis-related gene sets, including CASP10, CFLAR, and TNFRSF10A.

### MR analyses suggest alcohol consumption was associated with lower AMD risk

We considered the variants that remained significant after false discovery rate correction in both analyses as putative shared loci and included them in the final TSMR stage to assess directionality and clarify mechanistic links among gene expression, drinking behaviour, and AMD. We initially included 19 SNPs from the exposure GWAS. After evaluating the F-statistic, all 19 SNPs met the threshold of F>10 (mean = 6592.7; range = 130.5–40126.4) and were retained as final genetic instruments. The strong explanatory power of the selected SNPs for alcohol consumption ensures the validity of subsequent causal inference in MR analyses (Table S6 in the **Online Supplementary Document**).

We performed a TSMR analysis using 19 previously selected instrumental variants to evaluate the association between genetically predicted alcohol consumption and AMD (**Figure 2**, Panels A–C). The primary inverse-variance-weighted method indicated an inverse association (*β* = –0.13; *P* = 0.02). Similar protective effects were observed with the penalised weighted median (*β* = –0.10; *P* = 0.0003) and MR Egger (*β* = –0.24; *P* = 0.01) approaches. Maximum likelihood estimation suggested an even stronger inverse association (*β* = –0.30; *P* = 1.50 × 10^−10^). Across all methods, odds ratios consistently pointed to reduced AMD risk associated with a genetic predisposition to current alcohol consumption.

Sensitivity analyses, including leave-one-out and funnel plot assessments, confirmed the robustness of the MR findings (**Figure 2**, Panels D–F). We applied MR-PRESSO to detect and remove potential outlier SNPs (*P* < 0.05) and repeated MR analyses to account for horizontal pleiotropy (Tables S7 and S8 in the **Online Supplementary Document**). There was a substantial heterogeneity among the initial set of instrumental variables, which was markedly reduced after MR-PRESSO filtering. However, the causal estimates remained directionally consistent, indicating that the primary results based on the full set of 19 SNPs were not driven by outlier variants. There was no evidence of directional pleiotropy based on the MR-Egger intercept test, and the directions of effect estimates remained consistent across sensitivity analyses (Table S9 in the **Online Supplementary Document**).

There was no evidence of reverse causality – no methods were statistically significant for a causal effect of AMD on alcohol consumption (Table S10 in the **Online Supplementary Document**). Collectively, these results support the hypothesis that alcohol consumption may be associated with reduced risk of AMD, with consistent findings across multiple MR methods.

### The presence of rs3138141 is associated with increased retinal and RPE thickness in the UK Biobank

To explore potential structural correlates of the genetic instruments, we examined whether selected MR variants were associated with retinal OCT-derived phenotypes in the UK Biobank. Among the 19 instrumental variants, we evaluated three SNPs with available carrier counts and OCT-derived retinal measurements in the UK Biobank for associations with retinal thickness parameters. Carriers of rs3138141 showed significantly greater retinal thickness compared with non-carriers across multiple measures (all *P* < 0.001), including overall macular thickness and central subfield thickness in both eyes (Figure S5, Panels A–F in the **Online Supplementary Document**). Mean thickness values were consistently 2–3 μm higher in carriers, suggesting a potential effect of rs3138141 on retinal structure. Additionally, average RPE thickness was modestly increased in carriers (*P* = 0.02), further supporting a role for rs3138141 in modulating retinal architecture. In contrast, the other two SNPs (rs541862 and rs1265094) did not show statistically significant differences between carriers and non-carriers for any measured parameters.

### TNFRSF10A as a genetic and molecular link between alcohol consumption and AMD

We performed a colocalisation analysis using summary-level data from two GWAS data sets. Most variants showed low posterior probabilities for a shared causal variant (PP.H4), with the notable exception of rs13278062 near TNFRSF10A, which had a PP.H4 of 0.98 – well above the conventional 0.80 threshold for robust colocalisation. This suggests that this locus may harbour a shared causal variant influencing both alcohol-related traits and AMD risk (Table S11 in the **Online Supplementary Document**). The strong colocalisation at TNFRSF10A indicates that it is a potential key gene linking genetic susceptibility to AMD and warrants further investigation.

We analysed publicly available scRNA-seq data sets from human retina, RPE/choroid, and CD31-enriched RPE/choroid tissues from AMD patients to explore cell type-specific expression patterns (**Figure 3**, Panels A–D). In two independent data sets, TNFRSF10A was mainly expressed in endothelial cells, with about 20% of cells showing detectable expression and relatively high average levels. In a third data set, enriched for endothelial cells and subdivided into artery, choriocapillaris, and vein populations, TNFRSF10A was preferentially expressed in venous endothelial cells. Expression in other cell types was minimal, supporting its potential role in AMD-related vascular and endothelial processes. In the UK Biobank (current drinkers), Olink plasma proteomics showed significantly lower TNFRSF10A levels in individuals with AMD compared with non-AMD controls (*P* = 0.02) (**Figure 3**, Panel E). AlphaFold modelling indicated a high-confidence structure for TNFRSF10A. Molecular docking suggested possible binding sites for drugs such as troglitazone, pentoxifylline, paclitaxel, and alvocidib, providing a structural basis for future exploration of pharmacological modulation (Figure S6 and Table S12 in the **Online Supplementary Document**).

## DISCUSSION

By integrating epidemiological evidence from large cohorts with genetic instruments and molecular expression data, we evaluated the relationship between alcohol consumption patterns and the risk of AMD, as well as the potential mechanisms involved. Among current drinkers, overall alcohol intake had an inverse association with AMD risk, an association largely driven by wine consumption. By contrast, beer consumption had a positive association with AMD risk. MR analyses provided genetic support for these associations. Multi-omics integration further identified the TNFRSF10A locus as a shared genetic determinant of both alcohol intake and AMD susceptibility.

Although GWAS have identified >100 loci linked to AMD, clarifying the causal genes and functional mechanisms remains difficult. This difficulty is due to linkage disequilibrium and the fact that most variants lie in non-coding regions [29,30]. In this study, we used a different approach by combining large summary-level GWAS data sets for alcohol consumption and AMD with blood cis-eQTL information. Using SMR in combination with colocalisation analyses, we evaluated genetic variants in data from over one million individuals of European ancestry and identified signals shared by both traits. This analysis goes beyond mere statistical links, highlighting variants that may be associated with both alcohol use and AMD through genetically regulated gene expression. The rs13278062 near TNFRSF10A showed strong colocalisation (PP.H4 = 0.98), pointing to a shared causal variant. This SNP is highly expressed in endothelial cells, especially in retinal veins and RPE/choroid tissue, suggesting the potential roles in endothelial dysfunction and inflammation. These results point to genetic pathways where gene expression, drinking behaviour, and AMD risk intersect, providing a basis for further mechanistic studies.

Epidemiological analyses showed that overall alcohol and wine consumption at lower-to-intermediate levels were inversely associated with AMD risk in a broadly linear manner, whereas beer consumption displayed a nonlinear dose-response relationship with increased risk. This aligns with some previous observational studies, though other reports have found no association or even potential harm [4–10,31,32]. Such heterogeneity may stem from differences in study design, how alcohol types are categorised, residual confounding (*e.g.* socioeconomic status or overall lifestyle), and ‘sick quitter’ bias. By limiting our primary analysis to current drinkers and using MR, we could address some of these challenges. Estimates from multiple MR approaches suggested an inverse association, and sensitivity analyses showed no evidence of substantial horizontal pleiotropy, supporting a potential genetic association between alcohol intake and AMD risk. Nonetheless, it is important to emphasise that no study advocates alcohol consumption as a preventive strategy for AMD, given the potential for bias, confounding, reverse causality, and the broader health risks associated with drinking.

Notably, different types of alcoholic beverages appear to have distinct effects. Our observation of an increased risk for beer drinkers is consistent with previous studies [4,33], which link beer consumption to the incidence of soft indistinct drusen, drusen area, and confluent drusen in men [33], suggesting that beer may have distinct mechanistic pathways compared to other alcoholic beverages. In contrast, red wine contains polyphenols that have been shown to reduce cardiovascular risk through enhanced nitric oxide-mediated vasodilation, inhibited platelet aggregation, and anti-inflammatory effects, mechanisms that may also be relevant to retinal vascular health [34]. The observed differences between beer and other alcoholic beverages may also reflect lifestyle and socioeconomic factors. Wine consumption is often associated with healthier behaviours and higher socioeconomic status, which can reduce AMD risk, whereas beer intake tends to correlate with less healthy habits, such as smoking and high-fat diets, which can increase AMD risk [35].

This lifestyle-related confounding may also be affected by selection bias inherent to our primary data source, the UK Biobank. UK Biobank participants tend to be healthier, more socioeconomically advantaged, and more likely to engage in health-promoting behaviours, such as wine consumption combined with balanced diets [36,37]. This ‘healthy volunteer effect’ may exaggerate the apparent protective association between wine and AMD, as the observed benefit may partly reflect the baseline health advantages of wine drinkers in the cohort rather than a direct biological effect of wine itself. A previous MR study reported a harmful effect of alcohol consumption on advanced stages of AMD [12]. In contrast, genetic evidence supported an association between alcohol intake and reduced AMD risk among current drinkers, helping to address the abstainer bias commonly encountered in alcohol epidemiology [38]. These findings were consistent across multiple sensitivity analyses. However, the generalisability of the results to more diverse or less healthy populations may still be limited due to the characteristics of the UK Biobank cohort [36,37]. Despite the genetic evidence suggesting an inverse association, the well-established and widespread harms of alcohol consumption across multiple health domains far outweigh any potential, modest benefit for AMD. Thus, our findings should be interpreted solely as providing new biological insights into disease pathogenesis and should under no circumstances be taken as a justification for alcohol consumption.

Colocalisation analyses highlighted TNFRSF10A, which encodes the tumour necrosis factor-related apoptosis-inducing ligand receptor 1 (TRAIL-R1/DR4), a key regulator of apoptosis and inflammatory responses. Using scRNA-seq, we found that TNFRSF10A is predominantly expressed in endothelial cells in the retina and choroid of AMD patients. Functional enrichment analyses further pointed to immune regulation and apoptosis-related pathways [39,40], leading us to propose a scientific hypothesis: alcohol intake, or specific compounds in wine, may down-regulate TNFRSF10A-mediated signalling, reduce the release of proinflammatory cytokines (*e.g.* TNFα, IL-6), attenuate caspase-mediated cell death, and inhibit NF-κB/MAPK activity. These effects may help preserve microvascular integrity, protect RPE from oxidative stress [41], and slow the progression of AMD. This hypothesis aligns with previous findings that TNFRSF10A contributes to endothelial apoptosis in Alzheimer disease and that grape polyphenols can suppress TNFRSF10A expression in inflammatory skin conditions [42], suggesting that this pathway may serve as a shared node linking nutritional factors, lifestyle, and age-related diseases.

### Limitations

This study has several limitations. First, in our primary analysis, we focused on current drinkers within the UK Biobank, which predominantly includes healthier individuals of European ancestry. This ‘healthy volunteer’ profile and the restriction to current drinkers may limit the generalisability of our findings to broader or more diverse populations. Second, data and model constraints, such as the exclusion of spirits and fortified wines, the absence of SNP rs13278062 in whole-exome sequencing data, and OCT thickness analyses limited to colocalised instruments, may restrict the scope of our conclusion, and residual confounding or bias related to exposure duration cannot be fully excluded.

Additionally, associations specific to different types of alcoholic beverages may be influenced by unmeasured lifestyle or cultural factors, which could partly explain the observed differences in AMD risk. Finally, the proposed role of TNFRSF10A in AMD pathogenesis remains hypothetical. Future studies using cell models, animals, and independent cohorts are needed to confirm its function and clarify the underlying biology, and some residual confounding may remain despite efforts to address it.

## CONCLUSIONS

By integrating multiple layers of data, we provide epidemiological and genetic evidence suggesting a potential association between alcohol consumption and AMD risk. The TNFRSF10A locus emerges as a plausible node linking drinking behaviour with AMD susceptibility. These observations offer a novel perspective on gene-environment interactions in AMD and generate a testable hypothesis involving vascular inflammation and apoptosis pathways in disease pathogenesis. Future research is warranted to explore this association in more diverse populations and to experimentally investigate the specific role of TNFRSF10A in retinal-choroidal vascular homeostasis and AMD progression, which could ultimately inform the development of targeted preventive or therapeutic strategies.

## Additional material


Online Supplementary Document
