# Supplementary material for: Genetic and epidemiological analyses of alcohol consumption patterns and age-related macular degeneration risk among current drinkers: a role for TNFRSF10A
Source: J Glob Health. 2026 May 22;16:04124. doi: 10.7189/jogh.16.04124 (PMC13196489; doi:10.7189/jogh.16.04124)
Supplement: Online Supplementary Document [file jogh-16-04124-s001.pdf]

Supplement to: Shen T, Li J, Yang X, Xia J, Zhou H, Ma Q, Wang Y, Wang J, Wang Z, Liu K, Yan B. Genetic and epidemiological analyses of alcohol consumption patterns and age-related macular degeneration risk among current drinkers: a role for TNFRSF10A. J Glob Health. 2026;16:04124.

Genetic and epidemiological analyses of alcohol consumption patterns and age-related macular degeneration risk among current drinkers: a role for TNFRSF10A

Table of content for supplement files

Supplementary Figures 2

Supplementary Tables 8

Supplement Methods 21

Supplementary Figures

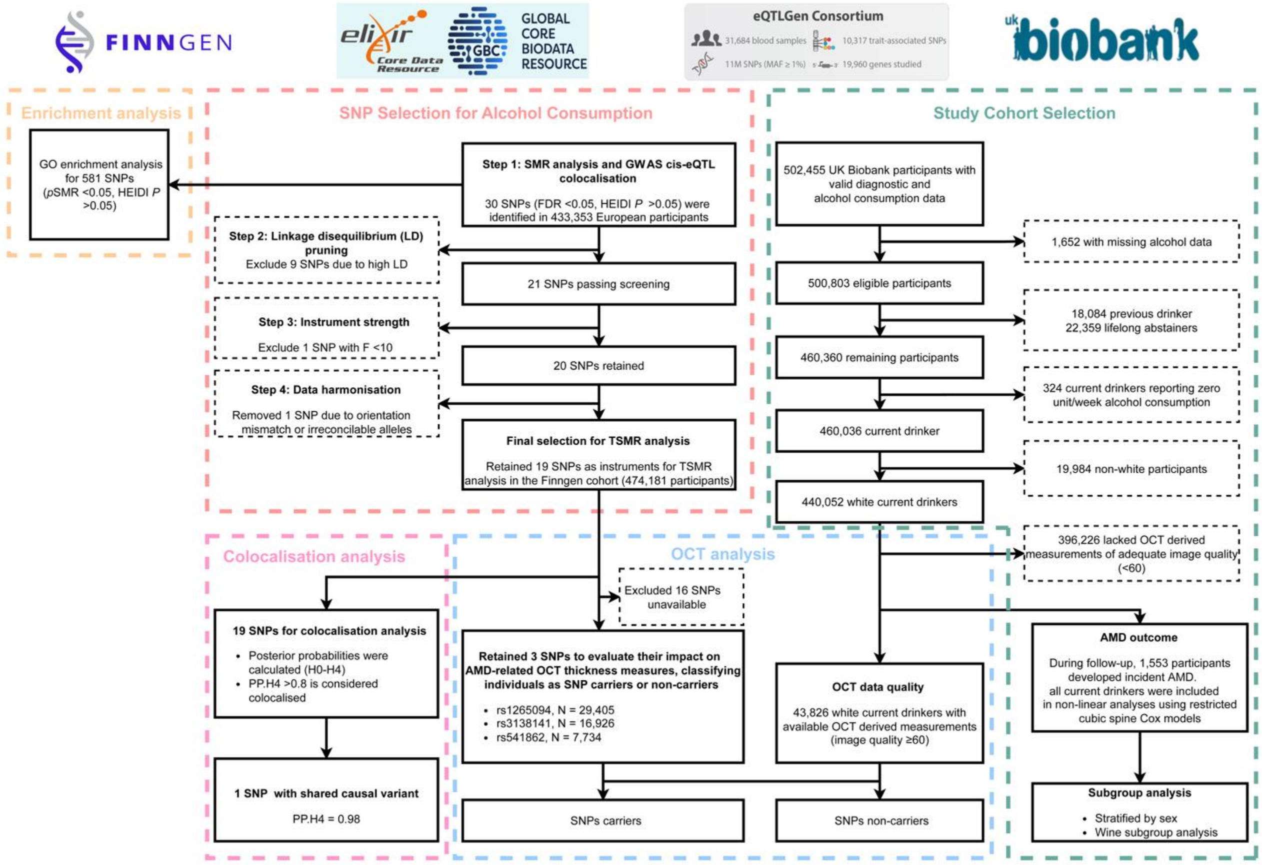

Figure S1. Flowchart of the selection process. AMD – age-related macular degeneration, cis-eQTL – cis-expression quantitative trait locus, GWAS – genome-wide association study, HEIDI – heterogeneity in dependent instruments, OCT – optical coherence tomography, SMR – summary-databased Mendelian randomisation, SNP – single-nucleotide polymorphism, TSMR – two-sample Mendelian randomisation.

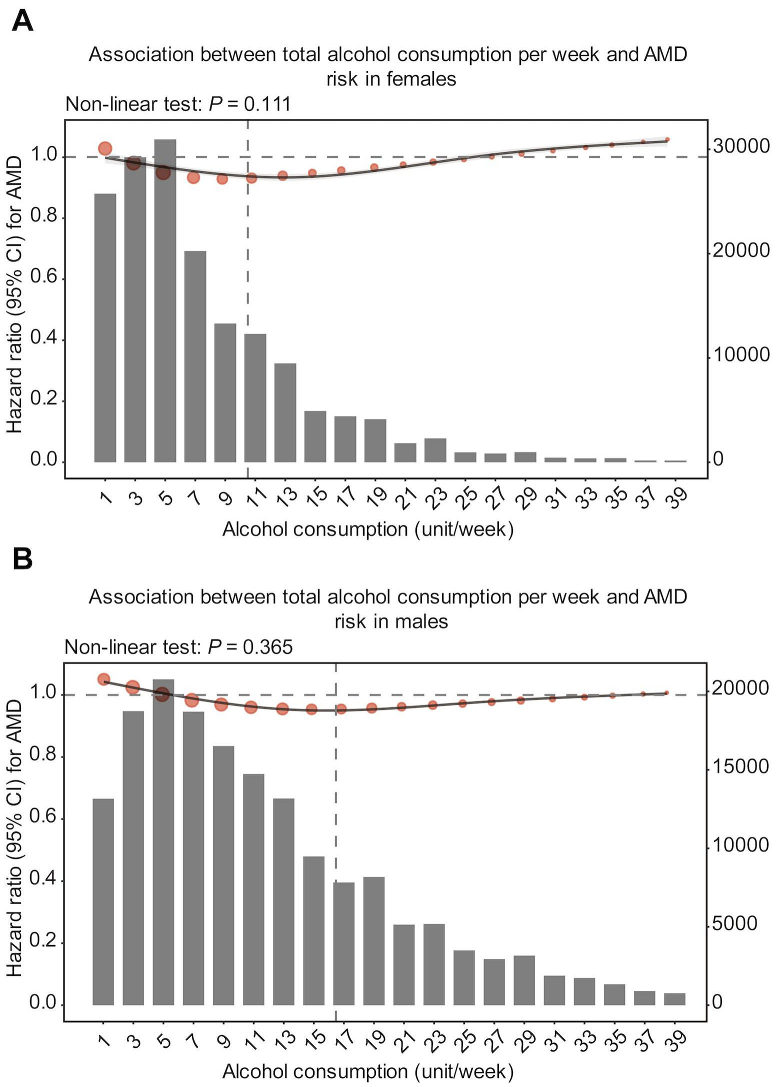

Figure S2. Dose-response relationships between alcohol consumption and age-related macular degeneration (AMD) incidence stratified by sex and alcohol consumption patterns. Panel A. Association between total alcohol consumption per week and AMD risk in females. **Panel B.** Association between total alcohol consumption per week and AMD risk in males. Hazard ratios (HRs) with 95% confidence intervals (CIs) were modelled using restricted cubic spline (RCS). Non-linearity was tested using a likelihood ratio test.

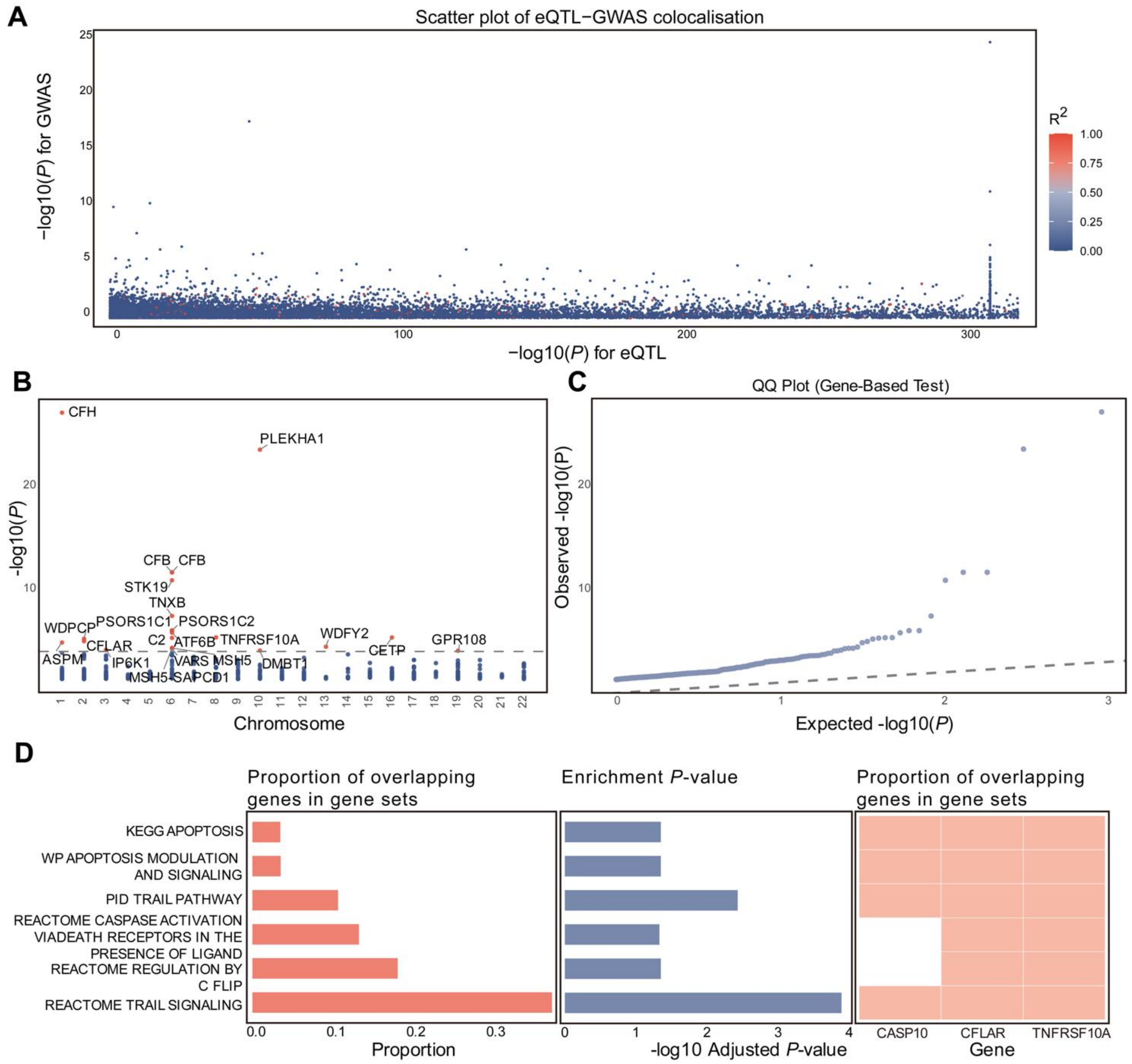

Figure S3. Cis-eQTL-GWAS colocalisation and Functional Mapping and Annotation of Genome-Wide Association Studies (FUMA-GWAS) results. Panel A. Scatter plot of eQTL-GWAS colocalisation. **Panel B.** Manhattan plot of gene-based associations from GWAS summary statistics. **Panel C.** QQ plot of gene-based associations from GWAS summary statistics. **Panel D.** Bar plots of the proportion of overlapping genes in gene sets. cis-eQTL – cis-expression quantitative trait locus, QQ – quantile-quantile.

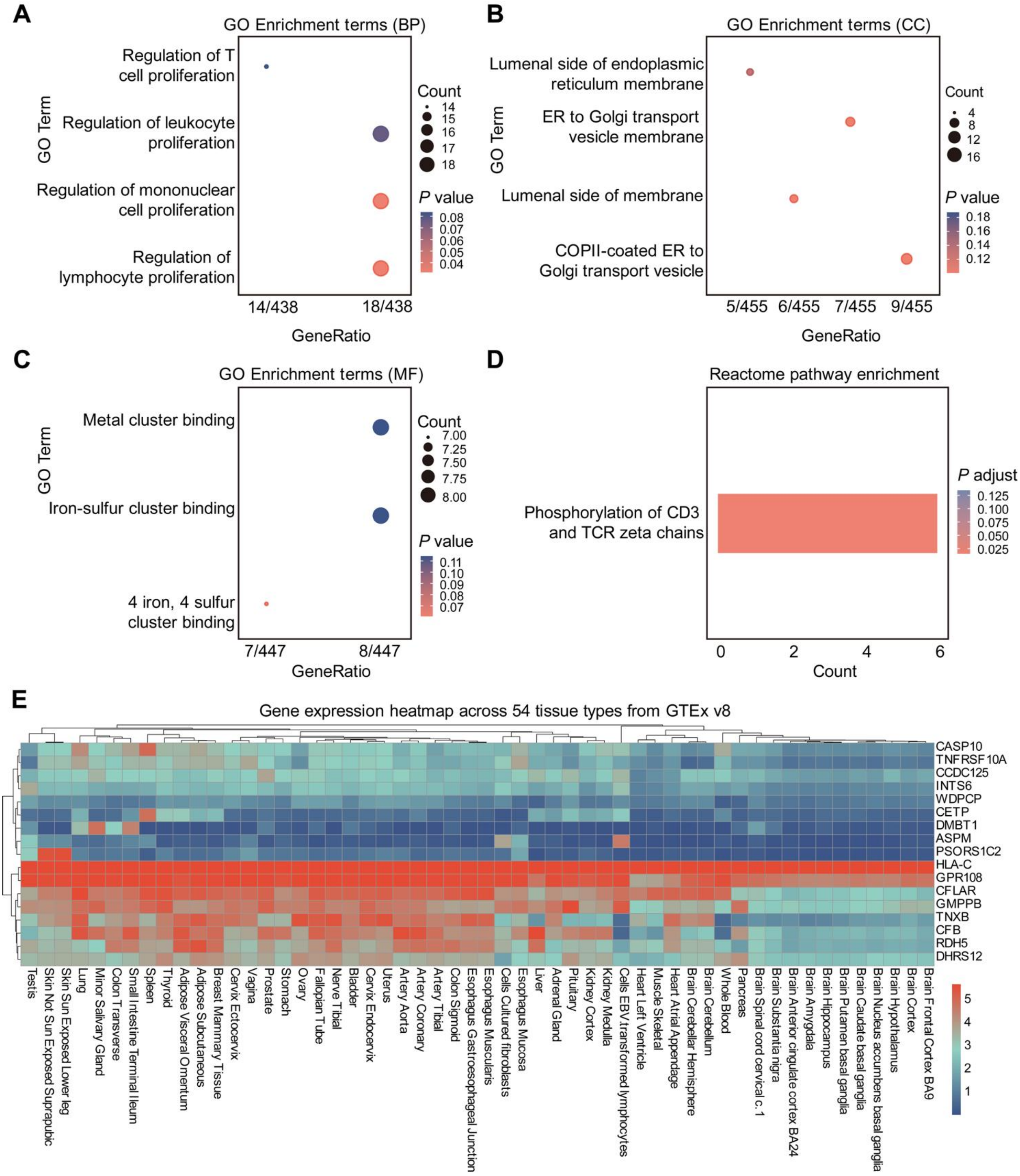

Figure S4. Top GO enrichment terms and Functional Mapping and Annotation of Genome-Wide Association Studies (FUMA-GWAS) results. Bubble plots show the top enriched GO terms. **Panel A**. Biological processes (BP). **Panel B.** Molecular functions (MF). **Panel C.** Cellular components (CC). **Panel D.** The top pathways in Reactome pathway analysis. The x-axis represents gene ratio, bubble size indicates gene count, and color reflects *P* value significance. **Panel**

E. Heatmap displaying the expression of key apoptotic genes across 54 tissue types from the GTEx v8 dataset.

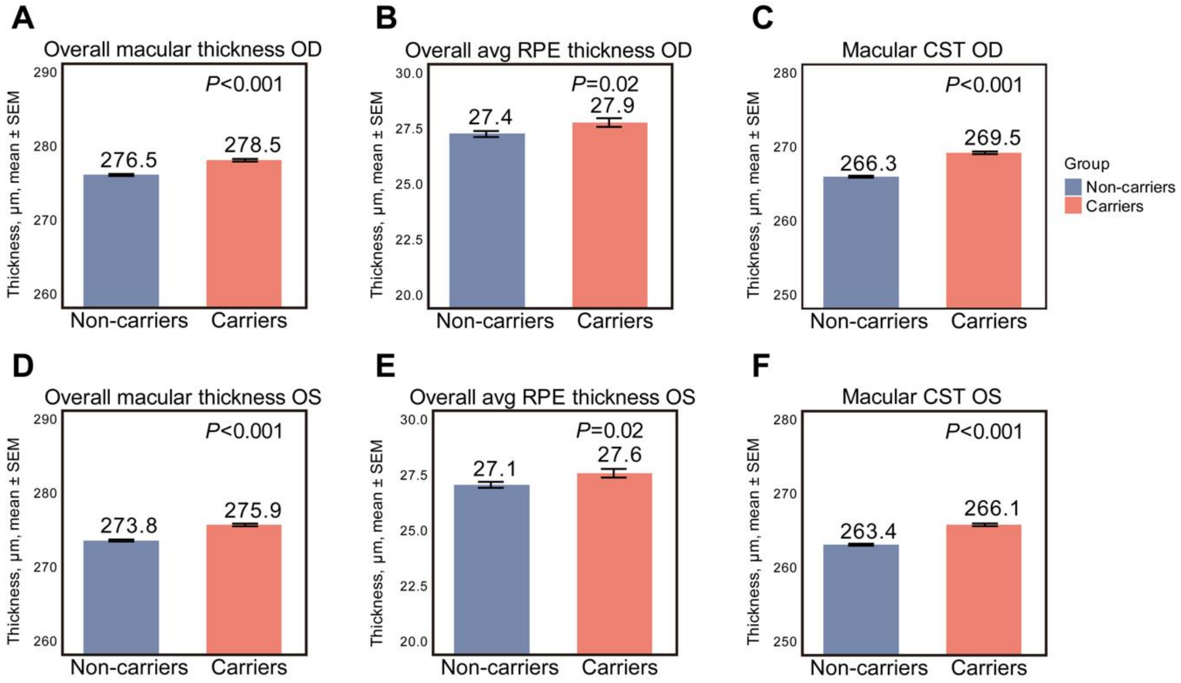

Figure S5. Effect of rs3138141 SNP carriage on OCT-derived retinal thickness measures. Bar plots comparing retinal thickness measurements in carriers and non-carriers of the rs3138141 SNP. **Panel A.** Overall macular thickness in the right eye. **Panel B.** Overall averaged retinal pigment epithelium (RPE) thickness in the right eye. **Panel C.** Macular central subfield thickness (CST) in the right eye. **Panel D.** Overall macular thickness in the left eyes. **Panel E.** Overall averaged RPE thickness in the left eye. **Panel F.** Macular CST in the left eye.

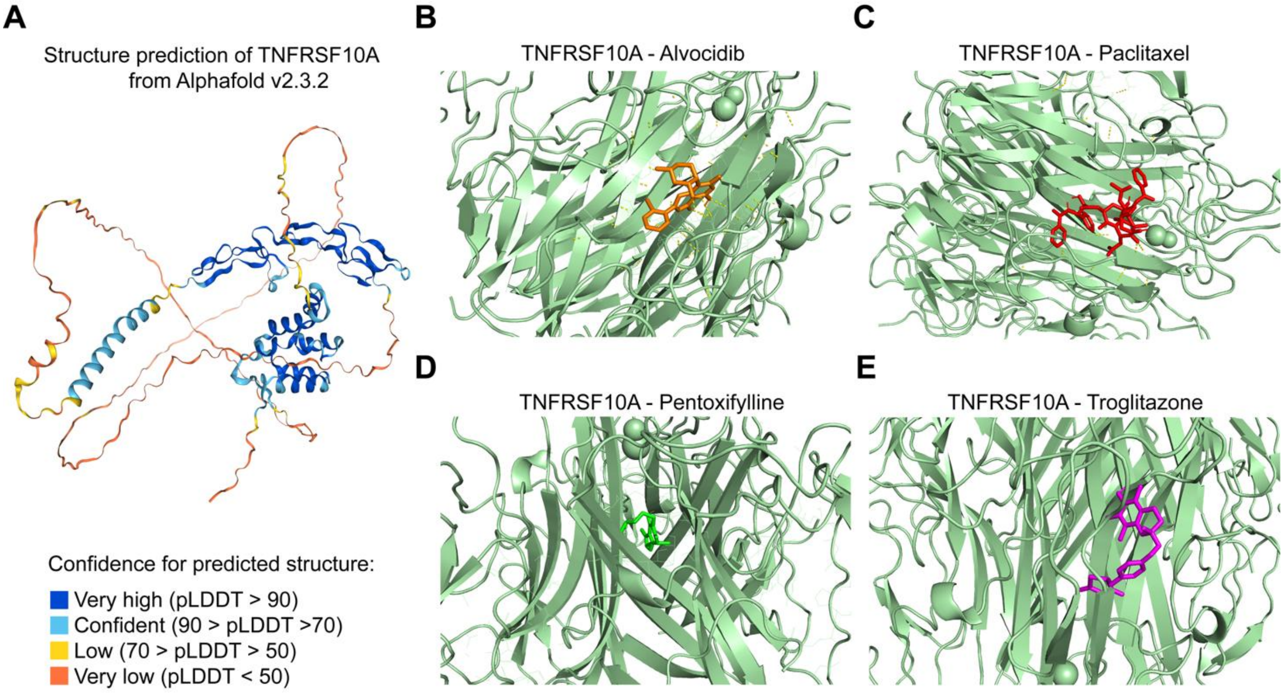

F

igure S6. Structure prediction and molecular docking interactions between small-molecule drugs and TNFRSF10A. Panel A. Structure prediction of TNFRSF10A generated by AlphaFold v2.3.2. Structure prediction image available from v24.0.proteinatlas.org. Docking models demonstrate the interactions between small-molecule drugs (colored ligands) and TNFRSF10A protein. **Panel B.** Alvocidib. **Panel C.** Paclitaxel. **Panel D.** Pentoxifylline. **Panel E.** Troglitazone. Ligands are visualized within the binding pockets of their respective targets, highlighting binding affinities and spatial orientation.

Supplementary Tables

Table S1. Outline of JoGH guideline items.

JoGH guideline item	Author's Response
1. Please list all papers published by each co-author in previous 3 years that were based on secondary analysis of a big data repository	1. Zhang, B., Li, J., Bai, Y., Jiang, Q., Yan, B., & Wang, Z. (2023). An improved microaneurysm detection model based on SwinIR and YOLOv8. Bioengineering (Basel, Switzerland), 10(12), 1405.<br>2. Wang, Z., Li, X., Yao, M., Li, J., Jiang, Q., & Yan, B. (2022). A new detection model of microaneurysms based on improved FC-DenseNet. Scientific reports, 12(1), 950.<br>3. Bai, Y., Li, J., Shi, L., Jiang, Q., Yan, B., & Wang, Z. (2023). DME-DeepLabV3+: a lightweight model for diabetic macular edema extraction based on DeepLabV3+ architecture. Frontiers in medicine, 10, 1150295.<br>4. Li, J., Ma, Q., Yao, M., Jiang, Q., Wang, Z., & Yan, B. (2024). Segmentation of retinal microaneurysms in fluorescein fundus angiography images by a novel three-step model. Frontiers in medicine, 11, 1372091.<br>5. Zhang, G., Qu, Y., Zhang, Y., Tang, J., Wang, C., Yin, H., Yao, X., Liang, G., Shen, T., Ren, Q., Jia, H., & Sun, X. (2024). Multimodal eye imaging, retina characteristics, and psychological assessment dataset. Scientific data, 11(1), 836.<br>6. Wang, Z., Zhong, Y., Yao, M., Ma, Y., Zhang, W., Li, C., Tao, Z., Jiang, Q., & Yan, B. (2021). Automated segmentation of macular edema for the diagnosis of ocular disease using deep learning method. Scientific reports, 11(1), 13392.<br>7. Zhu, Q., Liu, X., Qu, Y., Jiang, Y., Liu, X., Xiao, Y., Lv, K., Xu, Y., & Liu, K. (2025). Neuroprotective effects of healthful plant-based diets on retinal structure: insights from a large cohort. The journal of nutrition, health & aging., 29(1), 100431.<br>8. Wei, J., Xu, Y., Wang, H., Niu, T., Jiang, Y., Shen, Y., Su, L., Dou, T., Peng, Y., Bi, L., Xu, X., Wang, Y., & Liu, K. (2024). Metadata information and fundus image fusion neural network for hyperuricemia classification in diabetes. Computer methods and programs in biomedicine, 256, 108382.<br>9. Yang, X., Liu, Q., Ma, Q., Fan, X., Huang, C., Zhao, Y., Xia, J., Liu, T., Zhou, H., & Yan, B. (2025). Genome-wide Mendelian randomization study identifies therapeutic targets for diabetic microangiopathy. Diabetes research and clinical practice, 225, 112237.<br>10. Zhu, Q., Wang, H., Qu, Y., Jiang, Y., Liu, X., Liu, X., Xiao, Y., Lv, K., Xing, X., Niu, T., & Liu, K. (2025). Association between long-term serum uric acid variability and diabetic retinopathy: A prospective study in patients with Type 1 and Type 2 diabetes. Diabetes research and clinical practice, 226, 112306.<br>11. Zhu, Q., Liu, X., Qu, Y., Jiang, Y., Liu, X., Xiao, Y., Lv, K., Xu, Y., & Liu, K. (2025). Neuroprotective effects of healthful plant-based diets on retinal structure: insights from a large cohort. The journal of nutrition, health & aging, 29(1), 100431.
2. Please explain the key	This study leverages the UK Biobank cohort to investigate the relationship between

elements of your study design and the use of the available datasets that make your study an original scientific contribution	alcohol consumption and age-related macular degeneration (AMD) risk using advanced statistical and genetic methods. The definition of incident AMD ensures accurate case identification, and the focus on current drinkers minimizes sick-quitter bias, enhancing the reliability of the risk estimates. Non-linear associations between alcohol consumption and AMD risk were assessed using restricted cubic splines (RCS) Cox proportional hazards models, allowing for a more nuanced understanding of this relationship. Mendelian randomization (MR) was applied by integrating cis-eQTL and UK Biobank GWAS data to examine the causal effect of alcohol consumption on AMD. Genetic instruments were evaluated for strength using the F-statistic to ensure robust causal inference. This study provides novel insights into the genetic underpinnings of alcohol consumption and its potential impact on AMD risk.
3. Please list all publications that addressed similar research questions in the same dataset and indicate where you cited them in your paper	1. Xu L, You QS, Jonas JB. Prevalence of alcohol consumption and risk of ocular diseases in a general population: the Beijing Eye Study. Ophthalmology. 2009;116:1872-9. (Page 4 line 65)<br>2. Kuan V, Warwick A, Hingorani A, Tufail A, Cipriani V, Burgess S, et al. Association of smoking, alcohol consumption, blood pressure, body mass index, and glycemic risk factors with age-related macular degeneration: a mendelian randomization study. JAMA Ophthalmol. 2021;139:1299-306. (Page 5 line 82)<br>3. Su X, Wong TY. Revisiting the alcohol consumption association with age-related macular degeneration: what should we tell patients in 2021? JAMA Ophthalmol. 2021;139:1307-8. (Page 4 line 63)<br>4. Chong EW, Kreis AJ, Wong TY, Simpson JA, Guymer RH. Alcohol consumption and the risk of age-related macular degeneration: a systematic review and meta-analysis. Am J Ophthalmol. 2008;145:707-15. (Page 4 line 65)<br>5. Duic C, Vance E, Agrón E, Keenan TDL. Alcohol consumption and risk of age-related macular degeneration and geographic atrophy progression: age-related eye diseases study 2 report 34. Ophthalmol Retina. 2024. (Page 4 line 67)<br>6. Adams MK, Chong EW, Williamson E, Aung KZ, Makeyeva GA, Giles GG, et al. 20/20--Alcohol and age-related macular degeneration: the Melbourne Collaborative Cohort Study. Am J Epidemiol. 2012;176:289-98. (Page 4 line 65)<br>7. Knudtson MD, Klein R, Klein BE. Alcohol consumption and the 15-year cumulative incidence of age-related macular degeneration. Am J Ophthalmol. 2007;143:1026-9. (Page 4 line 65)<br>8. Fraser-Bell S, Wu J, Klein R, Azen SP, Varma R. Smoking, alcohol intake, estrogen use, and age-related macular degeneration in Latinos: the Los Angeles Latino Eye Study. Am J Ophthalmol. 2006;141:79-87. (Page 4 line 63)<br>9. Coleman AL, Seitzman RL, Cummings SR, Yu F, Cauley JA, Ensrud KE, et al. The association of smoking and alcohol use with age-related macular degeneration in the oldest old: the Study of Osteoporotic Fractures. Am J Ophthalmol. 2010;149:160-9. (Page 20 line 402)
4. Please explain how you addressed multiple testing	In our study, we addressed multiple testing by applying an appropriate False Discovery Rate (FDR) threshold during the summary-data-based Mendelian Randomization

through an appropriately rigorous statistical threshold and indicate this in the methods section	(SMR) analysis. Specifically, variants surpassing the FDR threshold were selected for further analysis in the two-sample MR (TSMR) to assess causal effects. Additionally, we used the standard statistical threshold for the F-statistic (>10) to retain strong genetic instruments and avoid weak instrument bias. These thresholds within the SMR and MR tools were utilized to ensure the robustness of our results and mitigate the risk of Type I errors.
5. Please declare to what extent have AI chatbots been used in developing your paper and to which parts of the paper did they contribute	AI chatbots were not used in the current manuscript.

Table S2. Study sample sources.

	Trait	Case N	Control N	Total N
**GCST90132924**	Alcohol drinker status: current vs never (UKB data field 20117)	433,353 European ancestry individuals	433,353 European (U.K.)	433,353
**GCST010723**	Early age-related macular degeneration	14,034 European ancestry cases, 91,214 European ancestry controls	105,248 European (Germany, NR)	105,248
**Finngen R12 H7 AMD**	Age-related macular degeneration (whether dry or wet)	12,495	461,686	474,181

Table S3. Baseline characteristics of overall current alcohol drinkers, with gender-based comparisons.

		Overall *	Female	Male	SMD
**N of participant**		440,052	236,106	203,946	NA
**Follow-up Years, mean (SD)**		12.9 (2.3)	12.0 (2.1)	12.75 (2.4)	0.10
**Alcohol consumption, unit/week**		11.5 (10.0)	9.0 (7.4)	13.95 (11.5)	0.51
**Alcohol Intake, N (%)**	**>14 units/week**	28,020 (6.4)	8,299 (3.5)	19,721 (9.7)	0.25
	**≤14 units/week**	412,032 (93.6)	227,807 (96.5)	184,225 (90.3)	
	**Missing**	0 (0%)	0 (0%)	0 (0%)	
**Age, years, mean (SD)**		56.65 (8.03)	56.4 (7.9)	56.9 (8.1)	0.07
**Age Group, N (%)**	**≤55 year**	321,312 (73.0)	175,099 (74.2)	146,213 (71.7)	0.08
	**[55, 65] year**	64,493 (14.7)	31,644 (13.4)	32,849 (16.1)	
	**>65 year**	54,247 (12.3)	29,363 (12.4)	24,884 (12.2)	
	**Missing**	0 (0%)	0 (0%)	0 (0%)	
**Socioeconomic, N (%)**	**Least deprived**	88,319 (20.1)	47,225 (20.0)	41,094 (20.2)	0.03
	**Middle deprived**	263,325 (59.9)	142,525 (60.4)	120,800 (59.3)	
	**Most deprived**	87,889 (20.0)	46,080 (19.5)	41,809 (20.5)	
	**Missing**	519 (0.1%)	276 (0.1%)	143 (0.1%)	
**Education, N (%)**	**Higher or vocational**	150,538 (39.8)	78,390 (38.5)	72,148 (41.4)	0.18
	**Other**	125,655 (33.2)	62,915 (30.9)	62,740 (36.0)	
	**Upper or lower secondary**	101,749 (26.9)	62,200 (30.6)	39,549 (22.7)	
	**Missing**	62,110 (14.1%)	32,601 (13.8%)	29,509 (14.5%)	
**BMI Group, N (%)**	**<18.5 kg/m^2**	2,085 (0.5)	1,656 (0.7)	429 (0.2)	0.35
	**[18.5-25.0) kg/m^2**	187,968 (42.9)	87,070 (37.0)	100,898 (49.7)	
	**[25.0-30.0) kg/m^2**	144,074 (32.9)	93,887 (39.9)	50,187 (24.7)	
	**≥30.0 kg/m^2**	104,096 (23.8)	52,632 (22.4)	51,464 (25.4)	

	**Missing**	1,829 (0.4%)	861 (0.4%)	968 (0.5%)	
**Ever Smoked, N (%)**	**No**	168,251 (38.2)	99,768 (42.3)	68,483 (33.6)	0.18
	**Yes**	270,387 (61.4)	135,591 (57.4)	134,796 (66.1)	
	**Missing**	1,414 (0.3%)	747 (0.3%)	667 (0.3%)	
**Sleep Duration Group, N (%)**	**[6-9] hours**	409,031 (93.0)	218,828 (92.7)	190,203 (93.3)	0.03
	**<6 hours**	28,835 (6.6)	15,874 (6.7)	12,961 (6.4)	
	**>9 hours**	2,186 (0.5)	1,404 (0.6)	782 (0.4)	
	**Missing**	4,825 (1.1%)	2,604 (1.1%)	2,221 (1.1%)	
**Physical Activity Group, N (%)**	**Additional**	46,158 (10.6)	17,020 (7.3)	29,138 (14.4)	0.25
	**Insufficient**	64,382 (14.8)	32,166 (13.8)	32,216 (16.0)	
	**Sufficient**	324,687 (74.6)	184,316 (78.9)	140,371 (69.6)	
	**Missing**	519 (0.1%)	276 (0.1%)	143 (0.1%)	
**Macular Thickness, µm, mean (SD)**	**OS**	266.8 (37.4)	262.7 (38.6)	271.48 (35.31)	0.24
	**Missing**	379,122 (86.8%)	203,499 (86.2%)	175,620 (86.1%)	
	**OD**	269.5 (37.5)	265.9 (38.6)	273.68 (35.78)	0.21
	**Missing**	379,119 (86.8%)	203,502 (86.2%)	175,620 (86.1%)	

[*] Percentages are calculated among participants with non-missing data for each variable unless otherwise stated. Missing values are reported where applicable. SMD indicates standardized mean difference.

Table S4. Univariate and multivariable Cox regression analyses of key potential confounders with age-related macular degeneration (AMD) risk.

	Parameter	Beta	SE	95% CI (Beta) Lower	95% CI (Beta) Upper	HR	95% CI (HR) Lower	95% CI (HR) Upper	*P* for interaction
**Education**	Higher Education	–0.028	0.056	–0.137	0.081	0.972	0.872	1.085	0.86
	Secondary Education	–0.098	0.064	–0.224	0.028	0.907	0.799	1.029	
	Vocational Qualifications	–0.110	0.117	–0.338	0.119	0.896	0.713	1.126	
**Townsend**	Least deprived	–0.047	0.054	–0.154	0.059	0.954	0.857	1.061	0.92
	Moderately deprived	–0.039	0.062	–0.160	0.082	0.962	0.852	1.086	
	Most deprived	–0.079	0.072	–0.220	0.061	0.924	0.803	1.063	
**Smoking**	Yes	–0.100	0.047	–0.193	–0.007	0.905	0.824	0.993	0.21
	No	–0.175	0.102	–0.374	0.025	0.840	0.688	1.025	
**Sleep**	<7 hrs	–0.148	0.078	–0.301	0.004	0.862	0.740	1.004	0.33
	[7,9) hrs	–0.015	0.040	–0.094	0.064	0.985	0.910	1.066	
	>=9 hrs	–0.538	0.242	–1.012	–0.064	0.584	0.363	0.938	
**BMI**	<25	–0.030	0.050	–0.128	0.067	0.970	0.880	1.070	0.11
	[25,30)	–0.060	0.057	–0.171	0.051	0.942	0.843	1.052	
	>=30	–0.342	0.132	–0.600	–0.083	0.711	0.549	0.920	
**Comorbidity***	Yes	–0.062	0.044	–0.149	0.024	0.940	0.862	1.025	0.99
	No	–0.052	0.077	–0.204	0.099	0.949	0.816	1.104	
**Full model†**	–	–0.180	0.072	–0.323	–0.037	0.835	0.724	0.964	–

* Comorbidity refers to the presence of any of the following comorbidities: diabetes mellitus (E10-E14), hyperlipidemia (E78), essential hypertension (I10), ischemic heart disease (I20-I25), asthma (J45), or arthropathies (M00-M25).

† Full Model: Cox regression model examining the effect of alcohol consumption (with 3 knots) on time to event, adjusted for sex, education, socioeconomic status, smoking, sleep, body mass index (BMI), and comorbidities.

Table S5. Cox proportional hazards analysis of alcohol intake and age-related macular degeneration (AMD) risk, stratified by beverage type and sensitivity analysis.

	Parameter	Beta	SE	95% CI (Beta) Lower	95% CI (Beta) Upper	HR	95% CI (HR) Lower	95% CI (HR) Upper	*P* (LR Test)	Reference Consumption (units/week)
**Total**	Alcohol	–0.181	0.067	–0.312	–0.050	0.834	0.732	0.951	0.11	17.2
	Sex	–0.105	0.050	–0.203	–0.007	0.900	0.816	0.993		
**Wine**	Alcohol	–0.148	0.062	–0.269	–0.026	0.863	0.764	0.974	0.08	9.1
	Sex	–0.127	0.049	–0.223	–0.032	0.880	0.800	0.969		
**Beer**	Alcohol	0.180	0.052	0.078	0.283	1.198	1.081	1.327	0.006	0
	Sex	–0.241	0.058	–0.354	–0.127	0.786	0.702	0.881		
**Fortified wine excluded**	Alcohol	–0.132	0.067	–0.263	–0.001	0.876	0.769	0.999	0.14	13.8
	Sex	–0.103	0.050	–0.201	–0.005	0.902	0.818	0.995		
**Spirits excluded**	Alcohol	–0.163	0.067	–0.293	–0.032	0.850	0.746	0.968	0.061	13.5
	Sex	–0.100	0.050	–0.198	–0.003	0.904	0.820	0.997		

Table S6. Characteristics of and strength evaluation metrics of genetic instruments for alcohol consumption.

SNP	Mapped Gene	Beta exposure	SE exposure	R^2	F	*P* value	FDR
rs1265094	PSORS1C1	0.48	0.10	0.115	14682.25	1.60E–10	0.007
rs13254617	TNFRSF10A	–0.11	0.02	0.006	630.76	2.34E–06	0.009
rs13413075	CFLAR	0.19	0.05	0.013	1454.81	1.66E–05	0.048
rs17201431	CFB	0.14	0.04	0.001	130.51	4.51E–05	0.045
rs17532524	WDFY2	0.16	0.04	0.007	800.88	7.39E–05	0.025
rs2269426	TNXB	0.16	0.03	0.013	1457.86	6.48E–06	0.003
rs2299851	MSH5	0.39	0.10	0.027	3184.46	5.06E–07	0.032
rs3019484	DMBT1	0.25	0.06	0.028	3192.86	2.30E–05	0.048
rs3138141	RDH5	–0.13	0.03	0.005	608.30	5.81E–06	0.001
rs339399	GPR108	0.32	0.08	0.051	6073.16	4.63E–18	0.048
rs35303934	HLA-C, HCG27	–0.11	0.03	0.003	289.36	5.62E–05	0.020
rs4976190	CCDC125	0.17	0.04	0.015	1693.15	8.95E–07	0.009
rs541862	CFB	0.46	0.06	0.034	3958.74	7.59E–06	2.00E–10
rs56228609	HERPUD1, CETP	–0.80	0.17	0.263	40126.38	4.78E–25	0.007
rs58679595	CFLAR	–0.17	0.04	0.015	1710.42	6.93E–05	0.018
rs6671696	ASPM	1.25	0.29	0.251	37682.77	3.59E–07	0.014
rs72889399	WDPCP	0.56	0.12	0.037	4297.22	2.32E–05	0.008
rs7374277	IP6K1	0.10	0.02	0.005	529.16	3.34E–05	0.044
rs9535673	INTS6-AS1	0.25	0.06	0.024	2758.51	3.29E–08	0.020

Table S7. MR-PRESSO outlier test results for the association between alcohol consumption and age-related macular degeneration (AMD) risk.

SNP	RSSobs	*P* value
rs1265094	1.52E–02	<0.019
rs13254617	5.21E–03	<0.019
rs13413075	1.09E–03	0.99
rs17201431	2.25E–02	<0.019
rs17532524	6.59E–04	0.99
rs2269426	1.19E–02	<0.019
rs2299851	9.45E–04	0.99
rs3019484	4.30E–03	<0.019
rs3138141	8.32E–03	<0.019
rs339399	1.03E–05	0.99
rs35303934	2.03E–03	0.17
rs4976190	2.02E–04	0.99
rs541862	1.41E–01	<0.019
rs56228609	1.93E–04	0.99
rs58679595	5.53E–03	<0.019
rs6671696	8.32E–02	<0.019
rs72889399	8.97E–03	0.13
rs7374277	4.86E–04	0.99
rs9535673	1.93E–03	0.15

Table S8. Mendelian randomization analysis of alcohol consumption and age-related macular degeneration (AMD) risk after MR-PRESSO outlier removal.

Method	N SNPs	Beta	OR	OR 95% CI Lower	OR 95% CI Upper	*P* value
MR Egger	10	–0.143	0.867	0.797	0.943	1.03E–02
Inverse variance weighted	10	–0.099	0.906	0.860	0.954	1.96E–04
Penalised weighted median	10	–0.115	0.892	0.841	0.946	1.34E–04
Maximum likelihood	10	–0.099	0.906	0.875	0.938	2.09E–08

Table S9. Heterogeneity and pleiotropy statistics for MR analysis of alcohol consumption and AMD risk.

Method	Value	*P* value
Inverse variance weighted Q (19 SNPs)	477.83	4.74E–90
MR Egger Q (19 SNPs)	409.52	1.89E–76
MR Egger intercept (19 SNPs)	0.05	0.11
Inverse variance weighted Q (10 SNPs)	30.33	0.0004
MR Egger Q (10 SNPs)	25.12	0.001
MR Egger intercept (10 SNPs)	0.02	0.23

Table S10. Mendelian randomization analysis of age-related macular degeneration (AMD) on alcohol consumption.

Method	N SNPs	Beta	OR	OR 95% CI Lower	OR 95% CI Upper	*P* value
MR Egger	8	0.004	1.004	0.995	1.012	0.43
Inverse variance weighted	8	–0.002	0.998	0.993	1.003	0.44
Penalised weighted median	8	–0.0004	0.999	0.995	1.004	0.85
Maximum likelihood	8	–0.002	0.998	0.994	1.002	0.35

Table S11. Summary of colocalization results between alcohol consumption and age-related macular degeneration (AMD).

Nearest gene	SNP.PP.H4*
CFB	0
TNFRSF10A	0.9834477
TNXB	0
CASP10	0
HLA-C	0
GMPPB	0
PSORS1C2	0
INTS6	0
WDPCP	0
DHRS12	0
CCDC125	0
CETP	0
GPR108	0
MSH5-SAPCD1	0
CFLAR	0
ASPM	0.9922814
DMBT1	0
RDH5	0

* SNP.PP.H4 represents the posterior probability for the lead SNP. A PP.H4 > 0.80 was considered evidence of robust colocalization. Genes were prioritized for further interpretation based on both statistical evidence of colocalization and biological relevance to AMD–related pathways.

Table S12. The binding energy between drug targets and the predicted drugs.

	Drug Targets	PDB ID	Predicted Drugs	PubChem ID	Binding energy (kcal/mol)
1	TNFRSF10A	5CIR	Troglitazone	5519	–8.2
2		5CIR	Pentoxifylline	4740	–7.5
3		5CIR	Paclitaxel	36314	–6.9
4		5CIR	Alvocidib	5287969	–8.7

Key resources table.

Resource	Source	Identifier
**Deposited data**		
UK Biobank data	UK Biobank	https://www.ukbiobank.ac.uk/
GWAS summary statistics	FinnGen	https://www.finngen.fi/en/access_results
GWAS summary statistics 2	GWAS Catalog	https://www.ebi.ac.uk/gwas/home
cis-eQTL summary data	eQTLGen consortium	https://www.eqtlgen.org/cis-eqtls.html
Single-cell RNA sequencing	Gene Expression Omnibus (GEO) repository	https://www.ncbi.nlm.nih.gov/geo/
Drug target genes	Drug Signatures Database	http://dsigdb.tanlab.org/DSigDBv1.0/
Protein structures	Protein Data Bank	http://www.rcsb.org/
Compound structures	PubChem Compound Database	https://pubchem.ncbi.nlm.nih.gov/
**Software and algorithms**		
R	The R Foundation	https://www.r-project.org
SMR	Zhu et al. 2016	https://yanglab.westlake.edu.cn/software/smr/#Overview
Docking engine	AutoDock Vina 1.2.2	http://autodock.scripps.edu/

Supplement Methods

Data Integration and Pre-processing

We relied on the European subset from the 1000 Genomes Project [1] to account for linkage disequilibrium (LD). Initial quality control entailed harmonising SNP alleles across datasets, excluding variants with excessive allele frequency deviations, and retaining SNPs that appeared in both the cis-eQTL and GWAS data. Only cis-eQTL associations with *P* values $<5.00\times10^{-8}$ were carried forward to ensure a stringent threshold for putative regulatory variants. Within each cis-eQTL-probe pair, SNPs with LD $r^2 < 0.05$ relative to the lead SNP were removed owing to insufficient linkage, and for SNP pairs with $r^2 > 0.90$, one SNP was removed to eliminate redundancy. The heterogeneity in dependent instruments (HEIDI) test was then performed to identify distinct causal variants in LD. SNP-probe pairs with a HEIDI *P* value > 0.05 were retained. *P* values for significant SNP-probe pairs were subsequently corrected for multiple testing using the Benjamini-Hochberg procedure, and associations with a false discovery rate (FDR) <0.05 were considered significant.

Two-Sample Mendelian Randomisation (TSMR)

The TSMR analysis incorporated summary statistics for AMD from the FinnGen study [2]. We harmonised the exposure and outcome data on shared variant identifiers and aligned effect alleles to maintain directional consistency. TSMR analyses were performed using inverse variance weighted (IVW), MR-Egger, penalised weighted median, and maximum likelihood methods. Heterogeneity was assessed via Cochran's Q, while MR-Egger intercept and MR-PRESSO were utilised to detect horizontal pleiotropy. A leave-one-out (LOO) analysis was conducted to identify any variants exerting disproportionate influence. We employed the same four classical MR methods (MR-Egger, Inverse Variance Weighted, Penalized Weighted Median, Maximum Likelihood) to analyze reverse causality (using AMD as exposure and alcohol consumption as outcome).

To evaluate the strength of genetic instruments and avoid weak instrument bias, we calculated the F-statistic for each SNP included in the exposure dataset (alcohol consumption). First, the coefficient of determination (R^2) for each SNP explaining the exposure variation was calculated using the formula: $R^2 = 2 \times (\beta_{exposure}^2) \times EAF_{exposure} \times (1 - EAF_{exposure})$, where βexposure represents the effect size of the SNP on alcohol consumption, and $EAF_{exposure}$ is the effect allele frequency of the SNP in the exposure GWAS (GCST90132924). Next, the F-statistic was derived based on the sample size (N = 433,353) of the exposure GWAS and R^2, using the formula: $F = (N - 2) \times R^2 / (1 - R^2)$. SNPs with an F-statistic > 10 (a commonly recognized threshold for strong instruments) were retained for subsequent MR analyses. All calculations of R^2 and F-statistic were performed using R software (Version 4.4.2) with the dplyr package for data processing, and the screening of qualified SNPs was conducted based on the F-statistic threshold of 10. To assess potential horizontal pleiotropy, we applied the MR-PRESSO method, which detects and corrects for outliers among the genetic instruments. SNPs identified as outliers ($P < 0.05$) were excluded in a sensitivity analysis to evaluate the robustness of the causal estimates.

Association between carriage of specific SNPs and macular thicknesses

We evaluated whether three single nucleotide polymorphisms (SNPs: rs541862, rs1265094, and rs3138141) were associated with OCT-derived retinal measurements (Topcon 3D OCT 1000 Mk2) from the UK Biobank cohort.

Colocalisation Analysis Methodology

In a ±150 kb region around each lead SNP, inconsistencies of effect alleles were resolved by swapping alleles or reversing effect estimates. We compiled effect sizes (β), variances (SE^2), minor allele frequencies (MAF, imputed to 0.5 if missing), sample sizes, and trait types from the binaryGWAS (case-control for both AMD and alcohol drinker status). We then applied the coloc.abf function, focusing on the five posterior probabilities (H0: no association with either trait; H1: association with trait 1 only; H2: association with trait 2 only; H3: both traits associated but via distinct causal variants; H4: both traits share a single causal variant). SNP pairs with PP.H4 > 0.8 were considered to have strong evidence of colocalisation.

Protein structure prediction, candidate drug prediction and molecular docking

The predicted structure of TNFRSF10A, generated using Alphafold v2.3.2 [3], was obtained from the Protein Atlas database (v24.0.proteinatlas.org, https://www.proteinatlas.org/ENSG00000104689-TNFRSF10A/structure+interaction#proteinstructure [4]). Drug target genes identified based on instrumental variables from TSMR analysis were uploaded to the Drug Signatures Database (DSigDB, http://dsigdb.tanlab.org/DSigDBv1.0/) to identify potential therapeutic compounds, leveraging its extensive catalogue of gene-drug associations. Selected drug candidates were then further examined for their interaction with the encoded proteins via molecular docking. Protein and compound structures were obtained from the Protein Data Bank (http://www.rcsb.org/) and the PubChem Compound Database (https://pubchem.ncbi.nlm.nih.gov/), respectively. Water molecules were removed, and polar hydrogen atoms were added prior to docking. AutoDock Vina 1.2.2 (http://autodock.scripps.edu/) was employed to simulate protein-ligand binding, with grid boxes centred on each protein's structural domains. Binding energies (kcal/mol) were used to assess affinity, where values below −5.0 kcal/mol and −7.0 kcal/mol indicated good and strong binding, respectively.

Statistical methods

We examined a full model including alcohol consumption (with 3 knots), sex, education level, Townsend deprivation index, smoking status, sleep duration, BMI, and comorbidities (as defined by the presence of diabetes, hyperlipidemia, hypertension, ischemic heart disease, asthma, or arthropathies). We conducted sensitivity analyses stratified by socioeconomic status (Townsend deprivation index), comorbidities (diabetes, hypertension, cardiovascular disease), lifestyle factors (smoking, sleep duration, BMI), and education level (higher, secondary, vocational). Neither the full multivariable model nor stratified sensitivity analyses (by Townsend deprivation index, comorbidities, smoking, sleep duration, BMI, and education level) revealed a significant independent association between these covariates and AMD risk. As a result, they were not included as confounders in the primary Cox model, as their inclusion did not notably alter the main findings. Additionally, we performed interaction analyses to explore whether factors like alcohol consumption, sex, smoking status, sleep duration, BMI, comorbidities, education level, and Townsend deprivation index modify the relationship between alcohol consumption and AMD risk.

Reference

1. Consortium GP. A global reference for human genetic variation. Nature. 2015;526(7571):68. DOI: 10.1038/nature15393. [PMID: 26432245]

2. Kurki MI, Karjalainen J, Palta P, Sipilä TP, Kristiansson K, Donner KM, et al. FinnGen provides genetic insights from a well-phenotyped isolated population. Nature. 2023;613(7944):508-18. DOI: 10.1038/s41586-023-05837-8. [PMID: 36653562]
3. Jumper J, Evans R, Pritzel A, Green T, Figurnov M, Ronneberger O, et al. Highly accurate protein structure prediction with AlphaFold. Nature. 2021;596(7873):583-9. DOI: 10.1038/s41586-021-03819-2. [PMID: 34265844]
4. Uhlén M, Fagerberg L, Hallström BM, Lindskog C, Oksvold P, Mardinoglu A, et al. Proteomics. Tissue-based map of the human proteome. Science. 2015;347(6220):1260419. DOI: 10.1126/science.1260419. [PMID: 25613900]
